# Neutrophil Extracellular Traps: Potential Therapeutic Targets of Traditional Chinese Medicine and Natural Products for Cardiovascular Diseases

**DOI:** 10.3390/ph19010183

**Published:** 2026-01-20

**Authors:** Yichen Liu, Yunhe Guo, Xinru Wu, Peiyu Yan, Yan Wei

**Affiliations:** Faculty of Chinese Medicine, Macau University of Science and Technology, Macau 999078, China; liuyicc_@outlook.com (Y.L.); guoyhee@163.com (Y.G.); elysianwxr@163.com (X.W.)

**Keywords:** neutrophil extracellular traps, natural products, cardiovascular disease, potential targets, traditional Chinese medicine

## Abstract

Cardiovascular disease (CVD) remains a leading cause of global morbidity and mortality, and its initiation and progression are closely associated with multiple molecular mechanisms. Neutrophil extracellular traps (NETs) are mesh-like structures composed of DNA, histones, and antimicrobial proteins that are released by neutrophils during inflammation or infection. They play a crucial role in innate immune defense. However, when the dynamic balance of NETs is disrupted by excessive formation, persistent accumulation, or impaired clearance, NETs are no longer merely bystanders. Instead, they actively drive pathological processes in multiple CVDs and serve as a critical link between inflammation and cardiovascular injury. Given the central role of NETs in CVD pathogenesis, including atherosclerosis, myocardial ischemia–reperfusion injury, pulmonary arterial hypertension, atrial fibrillation, and heart failure, therapeutic strategies targeting NETs, such as inhibiting aberrant formation, enhancing clearance, or neutralizing toxic components, have emerged as promising approaches. In recent years, traditional Chinese medicine (TCM) and natural products have shown potential therapeutic value by modulating NET formation and promoting NET degradation, owing to their multitarget, multipathway regulatory effects. This article reviews the mechanisms by which NETs operate in CVDs and explores potential pathways through which TCM and natural active ingredients prevent and treat CVDs by regulating NETs. This review provides theoretical support for further research and clinical application.

## 1. Introduction

Cardiovascular disease (CVD) refers to a wide range of diseases affecting the heart and vascular system. Common CVDs include coronary heart disease, stroke, HF, and PAH [1]. According to statistics from the World Health Organization (WHO), approximately 17.9 million people die annually from CVDs, accounting for 31% of global deaths, indicating that CVDs pose a significant threat to public health worldwide [2]. The progression of CVDs involves complex cellular and molecular mechanisms, including oxidative stress, inflammation, apoptosis, endothelial dysfunction, and fibrosis. These mechanisms contribute to cardiac and vascular remodeling [3]. To develop effective preventative and therapeutic strategies, a deeper understanding of these molecular mechanisms is necessary. Currently, drug therapy and lifestyle modifications constitute the primary treatments for CVD. However, patient compliance is often poor due to the difficulty of consistently taking medication and maintaining dietary and exercise regimens [4]. Modern medicine has made significant advances in treating CVDs, yet traditional drug therapy continues to face numerous challenges, including substantial side effects and drug resistance. Therefore, developing safer and more effective treatments by identifying novel therapeutic targets is essential.

In recent years, research progress on NETs has provided an innovative approach for treating CVDs. In 2004, NETs were first described by Brinkmann et al. [5] as distinct from autophagy, apoptosis, and necroptosis. Unlike autophagy, apoptosis, and necroptosis, and distinct from classical phagocytosis and programmed cell death, NETs are extracellular networks released by neutrophils in response to pathogens or diverse stimuli. These structures consist of decondensed chromatin DNA decorated with histones and antimicrobial enzymes, including myeloperoxidase (MPO) and neutrophil elastase (NE). Their primary physiological function is to trap and eliminate pathogens and remove cellular debris, thereby supporting innate immunity [5,6]. Accumulating evidence suggests that NET formation is a double-edged sword. Excessive production or defective clearance leads to pathological accumulation of cytotoxic NETs in vascular walls and myocardial tissue. This process promotes CVD progression, including atherosclerosis, myocardial ischemia–reperfusion injury, diabetic cardiomyopathy, dilated cardiomyopathy, hypertrophic cardiomyopathy, myocardial infarction, and heart failure, through endothelial injury, coagulation activation, amplified inflammation, and fibrosis [7]. Therefore, modulation of NETs represents a promising therapeutic strategy for CVDs.

In recent years, traditional medicine, especially Chinese herbal medicine and natural products, has shown distinct value in preventing and treating CVDs. These therapies, characterized by multi-component, multitarget, and multi-pathway synergy, offer advantages in symptom improvement, delaying disease progression, and enhancing patients’ quality of life. They typically exhibit favorable safety profiles, with low toxicity and minimal side effects, thus garnering increasing attention and research interest [8]. Notably, some Chinese herbal medicines or natural products have demonstrated significant therapeutic potential against CVDs by regulating NETs. However, a systematic analysis or comprehensive review summarizing their roles in CVD treatment through NET regulation is currently lacking. This review systematically describes the biological characteristics of NETs and their critical roles in CVDs, emphasizing recent advances regarding Chinese herbal medicines and natural products targeting NET regulation. By integrating existing evidence, this article aims to identify potential active components, therapeutic targets, and associated pathways. Additionally, it critically discusses current research challenges and future development directions, providing a theoretical basis and scientific guidance for the development of novel NET-based cardiovascular therapeutic strategies.

## 2. Methods

This study employed a narrative review approach to explore neutrophil extracellular traps (NETs) as potential therapeutic targets of Chinese medicine and natural products for interventions in CVDs. We systematically searched the PubMed and Web of Science databases for literature published between 2010 and 2025. Keywords used were “NETs” or “NETs”; “CVD,” “atherosclerosis (AS),” “coronary heart disease (CHD),” “myocardial infarction (MI),” “heart failure (HF),” or “ischemia–reperfusion injury (I/R)”, or “pulmonary arterial hypertension (PAH)”; and “traditional Chinese medicine (TCM),” “Chinese herbal medicine,” “natural products,” or “phytochemicals.” Inclusion criteria covered randomized controlled trials, original research, observational studies, narrative reviews, systematic reviews, and meta-analyses. Exclusion criteria included duplicate publications, non-English articles, irrelevant studies, unpublished literature, and retracted papers. Articles that met the inclusion criteria after abstract screening underwent full-text evaluation to assess their quality and relevance.

## 3. Overview of NETs

Neutrophils are the most abundant immune cells in the human body. They act as the first responders against invading pathogens and play a pivotal role in pathogen clearance and immunoregulation [9]. When invaded by bacteria, fungi, viruses, or protozoa, neutrophils release a reticular and fibrous extracellular structure known as NETs [10]. Notably, NETs can capture and immobilize pathogens, hindering their spread and facilitating their removal. “NETosis” refers to the formation and release of NETs. Unlike apoptosis and necrosis, NETosis results from neutrophil activation and the release of decondensed chromatin, cytosolic components, and granule-derived proteins [11]. Currently, three primary forms of NETosis have been identified: suicidal NETosis, vital NETosis, and mitochondrial-driven NETosis. In this section, these types are described, and their distinct characteristics are compared.

Suicidal NETosis is the most classic and extensively studied pathway of NETs generation. It is mediated through an NADPH oxidase (NOX)-dependent mechanism, typically taking 2–4 h [12]. Specifically, neutrophils are activated by various stimuli, including PMA, CXCL8/IL-8, immune complexes, and microorganisms. These stimuli are recognized by surface receptors on neutrophils, such as G protein-coupled receptors, Fcγ receptors, and complement receptors. Receptor recognition triggers Ca^2+^ release from the neutrophil’s endoplasmic reticulum, resulting in increased intracellular Ca^2+^ levels [13]. Subsequently, protein kinase C (PKC) and the Raf-MEK-ERK-MAPK signaling pathway are activated, inducing NOX to produce reactive oxygen species (ROS). Accumulation of ROS promotes translocation of neutrophil elastase (NE) and myeloperoxidase (MPO) from azurophilic granules into the nucleus, where NE degrades histones. Simultaneously, further chromatin decondensation is facilitated by peptidyl arginine deiminase 4 (PAD4), which catalyzes the citrullination of nuclear histones [14,15,16]. Ultimately, the nuclear and granular membranes rupture, resulting in the mixture of chromatin and granular contents. Subsequently, the cell membrane ruptures, releasing NETs and leading to neutrophil death [17]. In the final stage of NET release, Gasdermin D (GSDMD) plays a crucial role in increasing membrane permeability, facilitating cell lysis and the extracellular release of NETs [18].

In 2012, Bryan and colleagues first reported that living polymorphonuclear neutrophils (PMNs) could rapidly release NETs in vivo, significantly differing from traditional suicidal NETosis involving cell death. This type of NETosis, which does not involve cell death, is termed vital NETosis [19]. Vital NETosis occurs within approximately 5–60 min, significantly faster than NOX-dependent suicidal NETosis (3–4 h). This process is primarily triggered by Ca^2+^ carriers and does not involve NOX [20]. During vital NETosis, chromatin undergoes decondensation and modification by granular proteins and histones. It is then incorporated into transport vesicles that bud from the nuclear membrane. These vesicles subsequently shuttle through the cytoplasm and fuse with the plasma membrane, without membrane perforation during the process [21]. The vesicles are then released extracellularly, forming NET structures, while the neutrophils remain viable. As this NETs generation mechanism allows neutrophils to continuously participate in immune surveillance and host defense, it is distinctly different from the NOX-dependent pathway.

**Figure 1:**
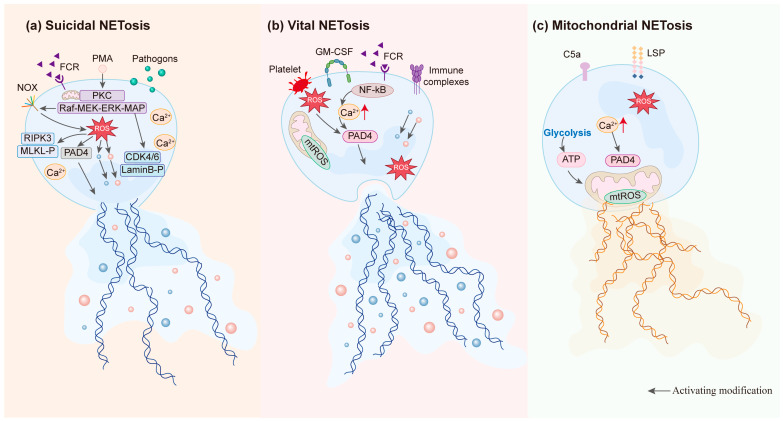
Three mechanisms for the formation of NETs.(**a**) Suicidal NETosis; (**b**) Vital NETosis; (**c**) Mitochondrial NETosis.

In addition to the classical NETosis pathway involving nuclear DNA, researchers discovered in 2009 that the release of mitochondrial DNA (mtDNA) can also generate NETs [22]. Pretreatment with granulocyte/macrophage colony-stimulating factor (GM-CSF) and stimulation of toll-like receptor 4 (TLR4) or complement factor 5a (C5a) receptors can mediate mitochondrial NET formation. Furthermore, ROS production plays a pivotal role in this process [23]. Mitochondrial NETosis occurs rapidly, typically within 15–20 min, distinguishing it from suicidal and vital NETosis [22]. Neutrophils activated by GM-CSF and C5a exhibit higher survival rates compared to resting neutrophils that do not produce NETs. This mechanism allows neutrophils to maintain nuclear integrity and retain other cellular functions. The triggering factors and distinct characteristics of this specialized NETosis pathway highlight the diversity of neutrophil responses under different physiological conditions [24] (Figure 1).

## 4. The Role of TCM and Natural Products in Treating CVDs by Regulating NETs

### 4.1. The Role of NETs in Myocardial Ischemia–Reperfusion Injury (MIRI)

MIRI occurs upon restoration of the blood supply after ischemia. It typically leads to cardiomyocyte death, cardiac dysfunction, fibrosis, and chronic cardiac impairment [25]. Accumulating evidence indicates that infiltration of inflammatory cells, such as neutrophils and macrophages, into the MI area is indicative of cardiac injury and represents a crucial pathological mechanism of MIRI [26]. During MIRI, activated neutrophils release numerous toxic substances, including ROS, proteolytic enzymes, and cationic antimicrobial peptides, directly causing pathological damage to myocardial tissue [27]. Furthermore, activated neutrophils secrete pro-inflammatory cytokines and chemokines, creating a positive feedback loop. This loop continuously recruits additional neutrophils to the injury site, intensifying local inflammation and ultimately causing an amplified inflammatory response [28].

**Figure 2:**
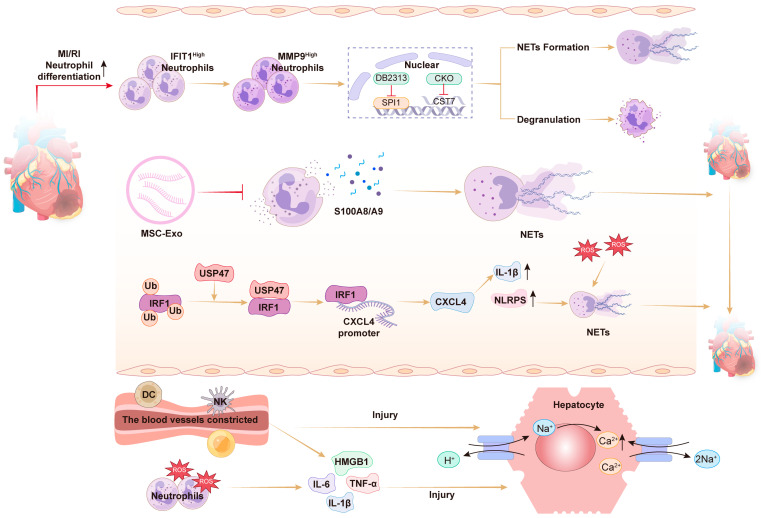
Mechanisms of neutrophil extracellular traps (NETs) formation in MIRI.

In addition, activated neutrophils secrete NETs, which consist of reticular DNA structures modified by histones and antimicrobial proteins [29]. Evidence suggests that NET formation contributes significantly to inflammatory damage in MIRI. For instance, NETs can promote ROS production and secretion of inflammatory cytokines and chemokines, thereby intensifying inflammation [16]. Similarly, inhibiting peptidyl arginine deiminase 4 (PAD4), a key enzyme responsible for NET formation, or degrading the NET scaffold with DNA enzymes can reduce damage caused by MIRI [6]. Studies indicate that accumulation of NETs and associated microthrombosis during myocardial ischemia (MI) or ischemia–reperfusion (I/R) impairs myocardial blood flow and exacerbates myocardial injury. Specifically, suppressing NETs and fibronectin significantly improves coronary microcirculation, reduces infarct size, and alleviates left ventricular remodeling after MI [30]. According to Feng et al. [31], mesenchymal stromal cell-derived exosomes (MSC-Exo) reduce neutrophil infiltration and NET formation following MIRI. Their beneficial effects occur by inhibiting activation of the NLRP3 inflammasome, ultimately decreasing microvascular obstruction (MVO) and preserving cardiac function post-MIRI [31]. Additionally, USP47 promotes pyroptosis via the NLRP3 pathway, exacerbating inflammation and impairing cardiac function after MI, with NETs involved in this process [32]. Yang et al. found that ALDH2 knockout activates the ER stress/Mgst2/LTC4 pathway, triggering NOX2-dependent NETosis and exacerbating MIRI in mice and patients [33]. Recent studies have shown that during MIRI episodes, neutrophils are rapidly activated via the SPI1/CST7 pathway and differentiate into a specific subpopulation characterized by elevated MMP9 expression. These neutrophils form harmful NETs, further exacerbating cardiac damage [34]. This evidence collectively indicates that NETs play a crucial role in MIRI pathophysiology, making them promising therapeutic targets (Figure 2).

### 4.2. The Role of TCM and Natural Products in Treating MIRI by Regulating NETs

TCM and natural products are important sources for drug discovery and treatment of CVDs. Numerous studies have shown that TCMs and natural products can alleviate ischemia–reperfusion (I/R) injury by modulating the balance of NETs. A hallmark of I/R injury is mitochondrial dysfunction associated with increased mitochondrial reactive oxygen species (mtROS) generation, directly triggering mitochondrial NETosis. Therefore, substances with mitochondrial-protective effects hold therapeutic promise in these circumstances. The modern Chinese herbal formulation Nuanxinkang, mainly comprising Red Ginseng and Mao Dongqing (Ilex pubescens), is traditionally prescribed to replenish Qi, enhance blood circulation, and promote detoxification. Research has confirmed that Nuanxinkang significantly reduces NET formation and ROS accumulation in mouse models of doxorubicin-induced myocardial injury. Its underlying mechanism may involve modulation of the NLRP3-HMGB1/IL-1β signaling axis, thereby suppressing NET formation and mitigating doxorubicin-induced cardiac damage. Within the tested dose range and study duration, no evident toxicity was observed in experimental animals [35]. Additionally, Qingxin Jieyu granules (QXJYF), another representative prescription developed by physician Chen Keji based on the “blood stasis-toxin interaction” theory for coronary artery disease, contain Huangqi (Astragalus membranaceus), Danshen (Radix Salvia miltiorrhizae), Chuanxiong (Ligusticum chuanxiong Hort.), Guanghuoxiang (Pogostemon cablin), and Huanglian (Coptis chinensis). Studies indicate that QXJYF improves cardiac performance and reduces myocardial pathological injury in rat myocardial infarction models, likely by activating the ANXA1/FPR2 signaling pathway to inhibit NET generation [36]. Preliminary animal studies did not detect significant toxicities under experimental conditions, but comprehensive toxicological evaluations are still necessary. Activated platelets are critical participants in myocardial ischemia–reperfusion injury (MIRI), and neutrophils are among the earliest immune cells recruited following MIRI. Upon activation, neutrophils release NETs, thus linking inflammation to thrombosis. Formononetin (FMN), a natural flavonoid isolated from herbs such as Astragalus membranaceus, Glycyrrhiza uralensis (Licorice), and Pueraria lobata (Kudzu root), inhibits platelet activation and NET formation via the CD36 pathway, thereby reducing thrombosis and inflammation in MIRI. This compound has demonstrated promising therapeutic effectiveness in animal models of I/R injury, along with favorable preliminary safety profiles, although further clinical studies are required to confirm its clinical utility [37]. Early neutrophil infiltration following I/R injury triggers oxidative stress, whereas NETs further promote activation of the NLRP3 inflammasome, subsequently inducing caspase-1-mediated NETosis [38]. Dunye Guanxinning (DG), an extract derived from the dried rhizomes of diosgenin-rich yam species (Dioscorea spp.), contains various saponins, including dioscin, gracillin, and diosgenin. Combined with traditional patented medications, DG has been reported to effectively reduce angina occurrence, recurrent myocardial infarction, cardiac insufficiency, and platelet activation in patients with acute myocardial infarction [39]. MPO production is essential for NET formation. Luo et al. [25] observed increased MPO activity in cardiac tissue of I/R rats, which was significantly reduced by pistachio consumption (30 mg/kg). These findings indicate that pistachios can inhibit NET formation, thus mitigating myocardial tissue damage [40]. Similarly, silibinin, a polyphenolic flavonoid derived from milk thistle or artichoke seeds, exhibits anti-inflammatory, antioxidant, anti-apoptotic, neuroprotective, cardioprotective, and lipid-regulating effects [41]. Research has shown that MPO-positive cells significantly increase in myocardial tissue following I/R injury in mice, an effect mitigated by silybin treatment, which reduces neutrophil infiltration and NET formation. However, human pharmacokinetic data for silybin remain limited, as its absorption, distribution, metabolism, and excretion may differ from those observed in animal models, potentially restricting clinical efficacy [42]. Panaxynol (PNN), also known as falcarinol, is a bioactive compound found in both medicinal and edible plants, including ginseng and carrot. It exhibits diverse pharmacological activities, including anticancer, antihypertensive, anticoagulant, neuroprotective, anti-inflammatory, immunomodulatory, and antibacterial properties [43]. For example, panaxynol not only decreases NET formation overall, but its derivatives can directly enter mitochondria, stabilize membrane potentials, and reduce mtROS leakage, thereby reducing myocardial infarct size and apoptosis [44] (Table 1).

### 4.3. The Role of NETs in AS

**Figure 3:**
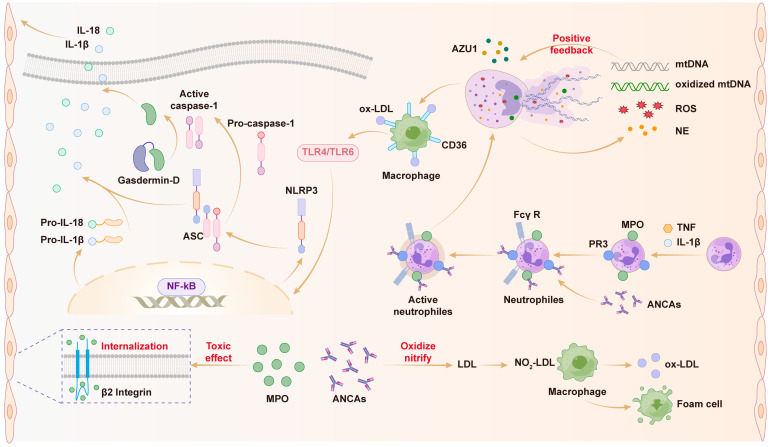
Mechanisms of neutrophil extracellular traps (NETs) formation in AS.

AS is an inflammatory disease primarily characterized by immune cell infiltration and cholesterol accumulation [45]. The progression of AS mainly includes three stages: fatty streak formation, induction of AS, and AS plaque development [46]. Increasing evidence suggests that neutrophils play key roles in AS plaque progression by forming NET structures, which have been identified in plaque tissues from humans and mice [47]. By inhibiting NET formation through deletion of PAD4 in neutrophils, AS plaques were reduced in nicotine-treated ApoE^-/-^ mice fed a high-fat diet (HFD) [48]. Shimonaga et al. reported elevated PAD4 levels in patients with unstable plaques compared to those with stable plaques [49]. PAD4 knockout in bone marrow-derived cells or administration of DNase I significantly reduced arterial intimal injury and plaque formation [50]. During plaque erosion, neutrophils in close contact with the inflamed endothelial surface undergo degranulation and ROS production, leading to endothelial cell death. Damaged endothelial cells expose prothrombotic factors, inducing platelet recruitment and aggregation, which further activate neutrophils and promote NET formation [51]. NETs released by activated neutrophils stimulate macrophages, facilitating the modification of LDL into ox-LDL, promoting foam cell formation, and sustaining AS progression. Another pro-inflammatory cytokine, macrophage-derived IL-8, is elevated in serum from patients with AS lesions. IL-8 interacts with CXCR2 on human neutrophils, promoting NET release and facilitating AS progression [52]. Furthermore, in advanced AS stages, proteolytic proteins (e.g., NE) derived from NETs, along with matrix metalloproteinases secreted by macrophages and T lymphocytes in AS plaques, damage plaque structure. This damage causes extracellular matrix leakage outside plaques, triggering thrombosis [53]. Recent studies revealed that excessive mitochondrial oxidative stress (mitoOS) increases oxidative damage to mitochondrial DNA (mtDNA), proteins, and lipids. Conversely, inhibiting mitoOS reduces 7-ketocholesterol-induced NET release from neutrophils in elderly mice [29]. Another study found that activation of the AIM2 inflammasome by NET-derived cell-free DNA (cfDNA) serves as an immune mechanism, activating MMPs, leading to plaque instability, AS thrombosis, and arterial embolism [54]. According to Geng et al. [55], TRAM acts as a stress sensor for oxLDL and free cholesterol during neutrophil inflammation. Defective TRAM expression reduces inflammatory mediator expression (LTB4 and elastase) and increases anti-inflammatory resolving mediators (RvD1 and CD200R), thereby inhibiting AS plaque progression [55]. Zhu et al. [56] found that inhibiting NET formation significantly reduces AS plaque formation and delays AS progression in animal studies. Additionally, low shear stress induces imbalanced intracellular Ca^2+^ concentrations and increases HDAC2 expression by downregulating Piezo1, thus promoting NETosis in cellular experiments [56]. In summary, compelling evidence suggests that NETs play a pivotal role not only in the formation and progression of AS lesions but also in AS thrombotic events (Figure 3).

### 4.4. The Role of TCM and Natural Products in Treating AS by Regulating NETs

TCM nd natural products exhibit targeted and multi-systemic effects in modulating NET formation, offering a novel approach for treating AS. For instance, 15,16-dihydrotanshinone I (DHT I), the most abundant diterpenoid tanshinone extracted from Salvia miltiorrhiza, has demonstrated efficacy in stabilizing vulnerable plaques and preventing thrombus formation in AS [57,58]. Quercetin, a natural flavonoid abundant in fruits, vegetables, and herbs, exhibits neuroprotective effects in patients with ischemic brain injury [59]. In an AS model, quercetin reduces MPO levels, p47phox expression, and NOX activity in blood vessels of ApoE^-/-^ mice, protecting vascular endothelial cells by inhibiting the MPO/H_2_O_2_ system [60]. Recent studies indicated that P2X7R is predominantly expressed in macrophages within AS plaques and is closely linked to the release of pro-inflammatory cytokines. Notably, P2X7R is identified as an upstream regulator of P38MAPK. Liu et al. [61] established an in vitro NET formation model by treating primary rat neutrophils with lysophosphatidylcholine (LPC). They found that quercetin reversed LPC-induced activation of the P2X7R/P38MAPK/NOX2 signaling pathway, consequently attenuating NET formation. However, high-dose quercetin exhibited potential nephrotoxicity in animal studies, possibly due to metabolite accumulation or oxidative stress-induced kidney damage; therefore, its toxicological profile needs further verification in clinical trials [61,62]. Collectively, current evidence suggests quercetin inhibits the early phase of NOX-dependent NETosis, thus reducing total NET production. Ferulic acid, a naturally occurring phenolic compound from Angelica sinensis and Ligusticum chuanxiong, exerts anti-apoptotic, anti-platelet aggregation, and antithrombotic activities. It is frequently used as adjunctive therapy in conditions like atherosclerosis and coronary artery disease [63]. Studies found that ferulic acid reduces vasculitis-related symptoms by inhibiting NET release and platelet activation, thus alleviating endothelial damage. Although this research does not directly address AS, it provides insight into potential AS treatment approaches through NET inhibition and platelet suppression [64]. Paeonol (PAE), an active compound isolated from peony bark, exhibits potent anti-inflammatory properties, effectively alleviating inflammation in AS [65]. Recent studies have shown that Pae effectively inhibits NE-mediated NET formation in plaques and foam cells in AS mouse models, disrupting CitH3, a key initiator of NET-associated inflammation. Thus, Pae reduces foam cell inflammation through the CitH3/NLRP3/caspase-1 signaling pathway, demonstrating anti-AS effects [66]. Additionally, TCM shows significant potential in treating AS. Modified Taohong Siwu Decoction (MTHSWD), an optimized form of the traditional Taohong Siwu Decoction (THSWD), comprises Prunus persica (Taoren), Carthamus tinctorius (Honghua), Angelica sinensis (Danggui), Ligusticum chuanxiong (Chuanxiong), Paeonia lactiflora (Baishao), and Rehmannia glutinosa (Shudihuang), supplemented with Astragalus membranaceus (Huangqi), cinnamon, and Epimedium brevicornum leaves. Shao et al. reported that MTHSWD possesses anti-inflammatory, antioxidant, anti-apoptotic, anti-platelet, anti-fibrotic, and lipid-lowering properties [67,68,69]. Recent findings by Shao et al. indicated that MTHSWD alleviates AS severity in mouse models, preserves vascular structure, enhances plaque stability, and maintains vascular patency, likely by reducing NE, MPO, and CitH3 expression. Moreover, treatment of human umbilical vein endothelial cells (HUVECs) with NETs demonstrated that higher concentrations and prolonged NET exposure caused more significant inhibition, damage, or apoptosis. Conversely, MTHSWD treatment promoted survival, growth, and proliferation of NET-injured HUVECs [70]. However, due to the multitarget and multipathway nature of TCM, the precise mechanisms of these therapies remain unclear, requiring additional research to clarify the effects of active components on AS. Regarding dose-effect relationships and long-term safety, TCM compound formulations demonstrate considerable dosage variability and generally favorable safety profiles. In summary, accumulating evidence supports the therapeutic efficacy of natural products and TCM in AS, suggesting promising prospects for clinical applications (Table 2).

### 4.5. NETs and PAH

**Figure 4:**
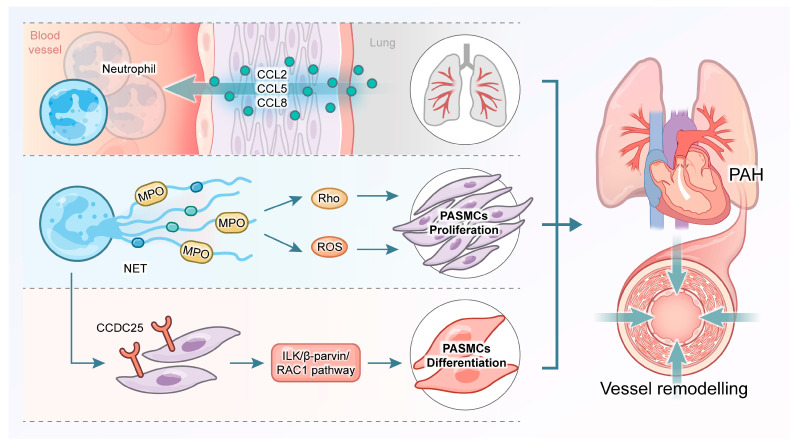
Mechanisms of neutrophil extracellular traps (NETs) formation in PAH.

PAH is a life-threatening disease characterized by the proliferation and remodeling of pulmonary arterioles. These changes result in elevated pulmonary vascular resistance and pulmonary artery pressure, ultimately causing right ventricular hypertrophy and right HF [71]. PAH involves complex pathophysiological mechanisms, including pulmonary artery smooth muscle cell (PASMC) hyperproliferation, pulmonary artery endothelial cell (PAEC) dysfunction, metabolic reprogramming, impaired angiogenesis, resistance to apoptosis, chronic inflammation, and phenotypic plasticity [72,73]. Recent evidence suggests that NETs, formed by neutrophil NETosis, contribute to the pathogenesis of PAH. For example, expression of NET-related markers is significantly elevated in lung tissue and plasma samples from PAH patients [74]. Additionally, the neutrophil-to-lymphocyte ratio (NLR) increases in PAH patients and correlates significantly with clinical deterioration and poor prognosis [75]. Vascular remodeling is a crucial feature of PAH. Components of NETs, such as MPO, stimulate PASMC proliferation through Rho kinase and ROS pathways, further promoting pulmonary vascular remodeling [76]. Moreover, NETs activate the transmembrane receptor CCDC25 on PASMCs, subsequently triggering the ILK/β-parvin/RAC1 signaling pathway. This activation leads to cytoskeletal remodeling and phenotypic transformation of PASMCs [77]. ET-1, a potent mitogen for vascular endothelial and smooth muscle cells (SMCs), acts as a vasoconstrictor linked to PAH pathogenesis. NET markers correlate significantly with ET-1 levels in plasma from chronic thromboembolic pulmonary hypertension (CTEPH) patients, suggesting ET-1 may regulate NET-induced responses [74]. MPO, an enzyme highly expressed in neutrophils, exhibits strong vasoconstrictive and profibrotic effects. Its expression increases in plasma and lung tissues of PAH patients, correlating with functional deterioration [78]. Furthermore, MPO^-/-^ mice exhibit resistance to hypoxia-induced PAH. Additional research indicates MPO enhances pulmonary vasoconstriction and SMC proliferation via Rho kinase, both in vivo and in vitro; notably, SMC proliferation is promoted by NET components [78]. During neutrophil recruitment to pulmonary regions, expression of chemokines such as CCL2, CCL5, and CCL8 increases, guiding neutrophil migration precisely to lungs during inflammation and thus contributing to PAH pathogenesis [79]. In summary, these findings highlight complex interactions among neutrophil migration, inflammatory mediators, and pulmonary vascular pathology. They provide a foundation for developing therapeutic strategies targeting specific inflammatory pathways and receptors. Such precise interventions may suppress neutrophil-mediated inflammation and facilitate favorable remodeling of pulmonary vascular structures, improving PAH treatment outcomes (Figure 4).

### 4.6. The Role of TCM and Natural Products in Treating PAH by Regulating NETs

TCM and natural products provide valuable resources for discovering novel chemical entities and developing more effective treatments for PAH. Due to their low cost and high safety, natural compound-based therapies have been used for thousands of years to enhance human health and are currently gaining increased attention. For instance, Huoxue Tongluo Formula (HXTLF), comprising Angelica sinensis, Carthamus tinctorius, Prunus persica, Paeonia lactiflora, Rubia cordifolia, Achyranthes bidentata, Lycopus lucidus, Citrus reticulata, and Cyperus rotundus, has properties of enhancing blood circulation, dredging collaterals, regulating menstruation, and relieving pain. Studies indicate that HXTLF modulates CitH3 and MPO protein expression in lipopolysaccharide (LPS)-activated neutrophils, thereby inhibiting NET formation and decreasing intracellular pro-inflammatory cytokines such as IL-1β. However, the specific bioactive serum components of HXTLF remain unclear, and its toxicological safety requires further clinical validation [80]. Chronic obstructive pulmonary disease (COPD), a critical risk factor for pulmonary arterial hypertension (PAH), involves excessive neutrophil activation within a chronic inflammatory airway environment. Neutrophils undergo classical lytic NETosis, releasing NETs and causing sustained airway injury, mucus hypersecretion, and tissue destruction. This process begins with massive ROS production triggered by NOX2 complex activation, subsequently activating MPO and promoting NE release from granules. Concurrently, ROS activates PAD4, inducing histone citrullination. NE and MPO then translocate into the nucleus, synergistically driving chromatin decondensation, eventually causing cell membrane rupture and extracellular NET release. These NETs form DNA-based networks enriched with histones and granule proteins, aggravating airway inflammation and lung parenchymal injury. According to Cheng et al. [81], Qingke Pingchuan (QKPC) granules, composed of Ephedra sinica, Prunus armeniaca, gypsum, licorice, Fagopyrum cymosum, Houttuynia cordata, Fritillaria cirrhosa, Ardisia japonica, Eriobotrya japonica leaves, and Perilla frutescens seeds, may alleviate neutrophil-driven airway inflammation, airway remodeling, and pulmonary dysfunction by reducing excessive NET formation. Its underlying mechanisms involve suppression of the NOX2/ROS signaling pathway, inhibition of NE and MPO activities, and regulation of PAD4-dependent histone modification. This provides a potential molecular immunological rationale for its clinical use in COPD and suggests possible future therapeutic applications in PAH [81]. Resveratrol, a natural polyphenol abundant in plants, exhibits anti-inflammatory, antioxidant, and anticancer properties. It suppresses phosphorylation and enzymatic activity of Src family kinases, mitigating endotoxin-induced lung injury by decreasing MPO levels, neutrophil infiltration, and neutrophil activation. These effects protect against PAH pathogenesis [82]. Grape seed proanthocyanidin (GSP), a flavonoid extracted from grape seeds, inhibits the progression of skin, lung, colon, and pancreatic tumors by regulating specific pathways and reducing oxidative stress. Additionally, GSP alleviates monocrotaline-induced PAH in rats by downregulating heat shock protein 70 (HSP70). An in vivo and in vitro study indicated that GSP reduces damage associated with PAH by inhibiting MPO expression and suppressing the proliferation of pulmonary artery smooth muscle cells (PASMCs) via TNF-α inhibition. These findings suggest GSP exerts therapeutic effects in monocrotaline-induced PAH rat models by inhibiting MPO activity and reducing inflammation [83]. Baicalin, the primary flavonoid isolated from Huangqin (Scutellaria baicalensis Georgi), possesses anti-inflammatory, antioxidant, antithrombotic, antitumor, and antiproliferative properties. Although direct evidence is lacking on its role in NET formation in PAH treatment, studies indicate that baicalin suppresses NET formation and citH3 expression induced by high glucose, LPS, or their combination, thereby maintaining intestinal epithelial integrity. Future research could investigate whether baicalin’s anti-inflammatory effects can therapeutically influence PAH by lowering pulmonary arterial pressure and preventing vascular remodeling [84,85].

Although direct evidence that zingerone, aloperine, and forsythiaside A can relieve pulmonary arterial hypertension (PAH) through modulation of NETs is currently unavailable, existing studies suggest potential mechanisms. Zingerone (ZIN), an active component of the traditional Chinese herb ginger (Zingiber officinale), significantly inhibits NET formation and inflammatory responses via the Nrf2-mediated reduction in ROS, providing a promising therapeutic strategy for sepsis-induced injuries [86]. FA, a primary compound extracted from the TCM Lianqiao (Forsythia suspensa), exhibits notable anti-inflammatory, antiviral, and antioxidant activities. Fan et al. reported that FA reduces NETosis in colon tissues, inhibits PAD4 expression in neutrophils, and suppresses PMA-induced NETosis in vitro [87]. Additionally, Wang et al. [87] identified that the classical and most extensively studied NET formation pathway involves NADPH oxidase (NOX)-dependent mechanisms. Aloperine (ALO), a quinolizidine alkaloid isolated from the traditional Chinese herb Sophora root (Sophora alopecuroides), demonstrates significant protective effects against PAH in monocrotaline (MCT)-induced rat models. Its therapeutic actions might involve suppressing NOX2 and NOX4 expression, thereby reducing oxidative stress [88]. These studies provide valuable insights for developing novel PAH therapies through NET regulation (Table 3).

### 4.7. NETs and HF

HF remains a leading cause of global morbidity and mortality, resulting in approximately 17.9 million deaths annually, and its burden continues to rise due to population aging [89]. HF is characterized by reduced physical activity, dyspnea, impaired left ventricular diastolic function, elevated left ventricular filling pressure, fluid and sodium retention, and abnormalities in systolic function, including impaired longitudinal strain and delayed myocardial untwisting [90]. The pathogenesis of HF is complex and multifactorial, often involving various inflammatory factors. Emerging evidence indicates that NETs contribute to the inflammatory environment in HF, promoting fibrosis and cardiomyocyte dysfunction. NETs may exacerbate HF progression by maintaining chronic inflammation and oxidative stress. This finding provides a new perspective for understanding the underlying mechanisms of HF. Although the specific mechanism linking NETs to HF remains underexplored, evidence suggests that neutrophil-related NETosis significantly contributes to myocardial tissue damage, fibrosis formation, and left ventricular remodeling in HF. Studies revealed increased circulating neutrophils and NET markers in patients with HF with preserved ejection fraction (HFpEF). Additionally, cardiac neutrophil infiltration and NET formation are elevated in HFpEF mice. Furthermore, HMGB1, an injury-related molecular pattern, is elevated in cardiac tissues of HFpEF mice. HMGB1 inhibition can reduce neutrophil infiltration and NET formation, thereby improving diastolic function [91].

**Figure 5:**
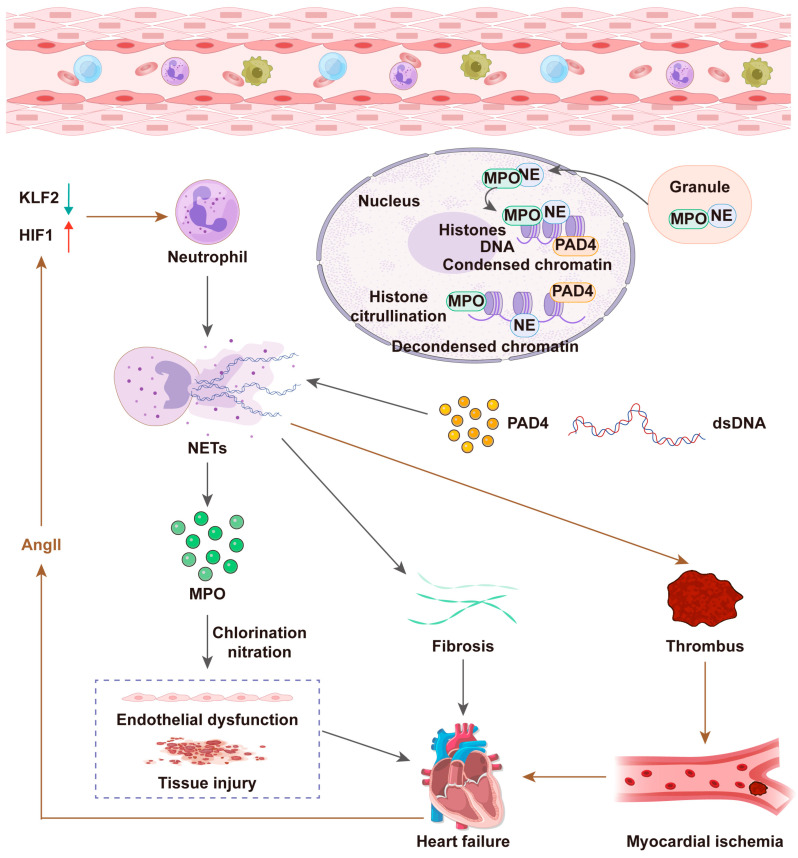
Mechanisms of neutrophil extracellular traps (NETs) formation in HF.

MPO, which primarily resides in neutrophils, plays a critical role in inflammation by catalyzing ROS formation, leading to cardiac remodeling, myocardial fibrosis, and endothelial dysfunction. Numerous studies demonstrate that elevated circulating MPO levels constitute an independent risk factor associated with HFpEF. MPO inhibition can attenuate inflammatory pathways linked to mortality, HF hospitalization, and impaired function in HFpEF patients [92,93]. Additionally, MPO is a predictor of the long-term prognosis of HF with reduced ejection fraction (HFrEF). MPO inhibition induces systemic anti-inflammatory effects and vasodilation, thus enhancing left ventricular function [94]. Recent research revealed a significant correlation between MPO and microvascular endothelial inflammation and injury, particularly prominent in HFpEF patients [95]. MPO may exacerbate HF pathology by facilitating biochemical reactions, including tyrosine chlorination and protein nitration, ultimately causing endothelial dysfunction and tissue damage [96]. In experimental studies using a BSCL2 gene-deficient mouse model, predisposed to developing HFpEF, researchers observed NET-mediated myocardial interstitial fibrosis. These results suggest that NETs contribute to disease progression by increasing ventricular stiffness, a characteristic pathological change in HFpEF. Furthermore, this study observed abnormal NET accumulation in myocardial tissue, exhibiting large, irregular morphological structures, supporting their potential role in cardiac pathology [97]. Studies reported that PAD4 gene knockout in mice diminishes neutrophil infiltration and NETosis induced by myocardial infarction [98]. Elevated NET levels were also observed in patients with HF and TAC mouse models. Both PAD4 knockout and NET inhibitors enhance cardiac function. Mechanistically, NETs induce mitochondrial dysfunction in cardiomyocytes by suppressing mitochondrial biogenesis via NE-TLR4-mediated inhibition of PGC-1α [99]. In summary, these findings support the hypothesis that NETosis participates in HF progression and represents a potential therapeutic target for future interventions (Figure 5).

### 4.8. The Role of TCM and Natural Products in Treating HF Through Regulating NETs

The role of TCMs and natural products in treating HF by modulating NETs has become an important research focus in cardiovascular medicine. NETs, as neutrophil-mediated immune defenses, may exacerbate HF progression. Excessive formation or impaired clearance of NETs can promote inflammatory responses, microthrombosis, and myocardial fibrosis. Due to their multitarget characteristics, TCMs and natural products potentially improve HF outcomes by regulating NET formation or degradation. For instance, mice with HF commonly exhibit chronic, low-grade systemic inflammation. Neutrophils, activated by inflammatory cytokines (e.g., IL-1β, TNF-α) or damage-associated molecular patterns (DAMPs), release NETs through pathways that are NADPH oxidase-dependent or independent. NETs, enriched with citH3, MPO, and NE, exacerbate inflammatory responses, subsequently damaging vascular endothelial and myocardial cells. Li et al. [100] reported that Nuanxinkang significantly reduces MPO and citH3 expression in cardiac tissues and peripheral blood of HF mice. This suggests that Nuanxinkang may inhibit inflammation by interfering with NET formation, thereby improving myocardial homeostasis and providing cardioprotection [100]. Since ROS directly influences NET formation, antioxidants have been explored as potential inhibitors of NETosis. Resveratrol (RSV), a polyphenolic compound abundant in many plants, has been shown to have anti-inflammatory, antioxidant, and antitumor properties. Its antioxidant mechanism primarily involves modulating NET release, essential for cardiovascular protection [101]. Recent studies indicate that RSV effectively reduces H2O2 levels in PMA-stimulated neutrophils and alters NE localization, thereby decreasing NET formation. This provides theoretical support for further assessment of RSV as a potential therapy for NET-associated diseases [102]. Geniposide (GE), an active component extracted from the dried mature fruits of Gardenia jasminoides Ellis, exhibits multiple pharmacological effects, including antitumor, neuroprotective, and hepatoprotective properties. GE regulates cardiac oxidative stress through the MMP2/SIRT1/GSK3β pathway, alleviating cardiac inflammation, apoptosis, fibrosis, metabolic disorders, and cardiac dysfunction in HFpEF. Although no studies currently address HFpEF treatment by targeting NETs directly, recent research indicates that GE promotes macrophage exocytosis by activating the AMPK-PI3K/Akt signaling pathway, clearing NETs, and improving acute kidney injury. Future research should explore whether GE can similarly improve HFpEF by modulating NETs [103,104] (Table 4).

### 4.9. NETs and AF

AF, the most prevalent arrhythmia, is characterized primarily by disordered atrial depolarization, causing symptoms such as palpitations and reduced exercise capacity. As the global population ages, AF has emerged as a significant health issue, with both incidence and prevalence increasing [108]. The onset and progression of AF are closely associated with inflammatory responses and immune cell activation. Neutrophils, a primary cell type in innate immune responses, release NETs, which contribute to AF development. For example, compared to patients with sinus rhythm, NET formation occurs in the left atrial appendage and coronary arteries of AF patients. NETs induce cardiomyocyte autophagic apoptosis and mitochondrial damage by promoting mitochondrial depolarization and ROS production. Prolonged tachycardia pacing damages cardiomyocyte structure and stimulates neutrophils to release NETs, creating a positive feedback loop that exacerbates AF progression [109]. Additionally, the epicardial adipose tissue (EAT) secretome in AF induces atrial fibroblasts to express ECM genes and contains high levels of MPO. In EAT, MPO levels rise before AF onset, with both MPO and NETs reaching peak concentrations in persistent AF, highlighting the role of EAT neutrophils in AF pathogenesis [110]. Another clinical study demonstrated that increased left atrial (LA) diameter and decreased left ventricular ejection fraction (LVEF) in AF are associated with enhanced NET formation [111]. These findings suggest that NETs participate in AF development, thus representing potential therapeutic targets.

MPO, a NET component, plays a crucial role in myocardial remodeling. Experimental and clinical studies have provided substantial evidence supporting the mechanistic role of MPO in AF pathophysiology. Liu et al. [112] reported elevated MPO expression in both paroxysmal and persistent AF, which positively correlated with left atrial volume (LAV). Elevated MPO levels may drive AF phenotypic transition and recurrence following catheter ablation [111]. Postoperative inflammation significantly influences postoperative AF (POAF), and MPO is a primary inflammatory mediator following surgical tissue injury. A clinical study of coronary artery bypass grafting (CABG) patients reported that postoperative AF incidence increased significantly with elevated MPO levels compared to controls, indicating MPO’s predictive role in postoperative AF [112]. Additionally, elevated levels of 3-chlorotyrosine, an active MPO metabolite, were detected in AF patients, demonstrating that MPO mediates oxidation reactions and protein modifications. These molecular effects contribute to atrial tissue structural remodeling. Mechanistically, MPO enhances matrix metalloproteinase (MMP) activity, exacerbating atrial fibrosis and electrical heterogeneity, core pathological features underlying AF onset and progression [113].

**Figure 6:**
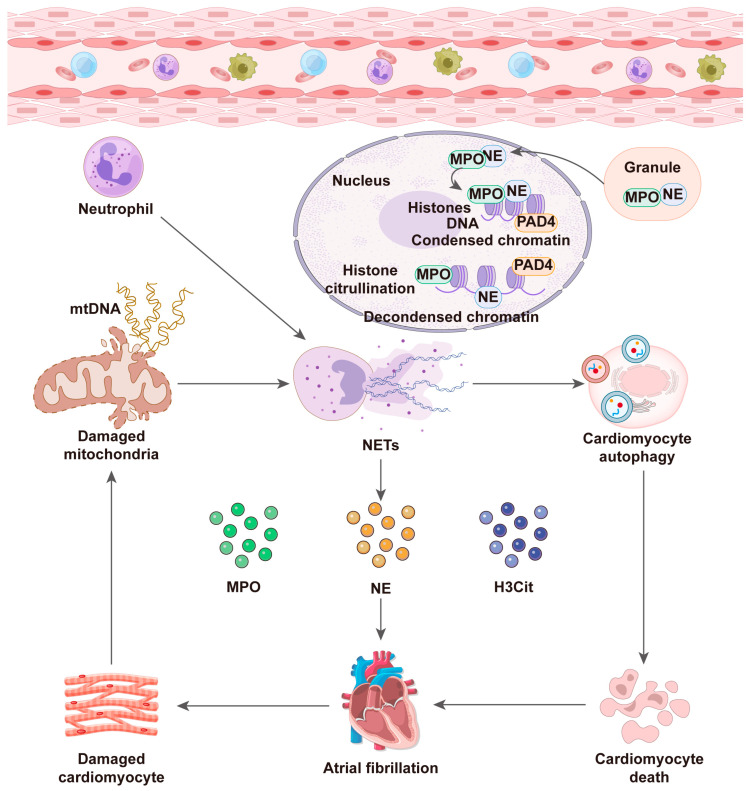
Mechanisms of neutrophil extracellular traps (NETs) formation in AF.

Moreover, elevated CitH3 levels in AF patients promote denser, more compact fibrin clot structures, reducing clot solubility and increasing thromboembolism risk [114]. Neutrophil activating peptide 2 (NAP-2, CXCL7), a novel fibrin clot regulator, is significantly elevated in AF and correlates with adverse fibrin clot structural abnormalities. Increased NAP-2 expression creates a prothrombotic microenvironment, exacerbated by synergistic neutrophil-platelet interactions [115]. Furthermore, S100A8/A9 expression increases in atrial tissues of chronic mild stress (CMS) mouse models, promoting atrial inflammation, NET formation, and activation of the TLR4-NLRP3 signaling pathway [105] (Figure 6).

### 4.10. The Role of TCM and Natural Products in Treating AF Through Regulating NETs

In recent years, research in cardiovascular medicine has increasingly focused on TCM and natural products for treating AF by modulating NETs. As key mediators of neutrophil immune response, NETs significantly contribute to various pathological processes in AF, including inflammation, thrombosis, oxidative stress, and atrial fibrosis. Tan et al. [105] reported that celastrol, a triterpenoid isolated from Leigongteng (Radix Tripterygium wilfordii), partly alleviates adverse arrhythmic effects following MI by promoting autonomic nerve, ventricular electrical, and ion channel remodeling. Additionally, celastrol improves ventricular fibrosis and inflammation by inhibiting the NLRP3/Caspase-1/IL-1β pathway. Additionally, celastrol inhibits PMA-induced NET formation in neutrophils in vitro, potentially by suppressing ROS production, offering theoretical insights into NET-associated disorders [105,106]. Kaempferol, a dietary flavonoid abundant in apples, strawberries, tomatoes, broccoli, green tea, and ginkgo leaves, possesses anticancer, anti-inflammatory, antioxidant, antidiabetic, neuroprotective, and cardioprotective properties. Given the strong association between AF progression and oxidative stress, and considering ROS as a classical trigger for suicidal NETosis, kaempferol inhibits ROS production in neutrophils, thereby effectively suppressing NET formation. By blocking ROS-induced NETosis, kaempferol exhibits potential antiarrhythmic and cardioprotective effects in AF, suggesting promising avenues for future research [107] (Table 4).

## 5. Discussion

In recent years, NETs and their role in CVDs have received increasing attention. Targeting structural components of NETs can effectively inhibit the onset and progression of CVDs. Although many studies have confirmed the significant role of neutrophils in CVDs, specific treatments targeting abnormal neutrophil activation remain limited. This limitation arises from two primary considerations. Firstly, directly altering neutrophil numbers or core functions could cause severe immunosuppression, significantly increasing infection risk. Secondly, an ideal pharmacological therapy should precisely target abnormal neutrophil function while preserving essential antibacterial capabilities. Consequently, this review summarizes the relationship between NETs and CVDs and explores strategies utilizing TCM and natural products to target NETs for treating CVDs.

However, significant challenges remain for the clinical application of these findings. Firstly, neutrophils have a short lifespan and are readily activated or degraded by various physiological and non-physiological NET activators. This variability leads to diverse effects and activation of multiple pathways. Secondly, compared with healthy controls, NET levels significantly increase in various CVDs, including coronary heart disease, AF, PAH, MIRI, and HF. Furthermore, NET levels display heterogeneity and complexity due to differences in disease origins, molecular biomarkers, and immune microenvironments. Consequently, RNA scoring systems based on NETosis-related gene expression might not accurately reflect the formation status of NETs in the cardiovascular microenvironment. This discrepancy results from diverse NET biomarkers, such as NET-DNA, CitH3, NE/NE-DNA, and MPO/MPO-DNA. Significant differences in NET levels may arise when using different markers or even the same marker with varying detection methodologies. Clearly, establishing standardized quantitative methods for NET detection is essential for clinical applications.

Additionally, NETosis is not a single-step process but rather a dynamic process lasting approximately 220 min. However, research on the dynamic changes during this period remains limited. Moreover, different cell types form NETs differently when triggered by various stimuli, exerting distinct impacts on CVDs at different stages. Currently, most experimental studies are conducted on animals or cell models, and few formal clinical trials specifically target NETs in human CVDs. Thus, existing studies may not fully represent human disease progression or accurately assess the effects of NETs. Consequently, therapeutic targets derived from experimental studies require validation for feasibility and safety in humans [116]. Lastly, the molecular mechanisms underlying NET formation remain incompletely understood, particularly regarding the precise activation of PAD4 and its synergistic role in NET release. Precisely regulating NET formation and function, and translating these insights into clinical interventions, remains challenging. Future research should focus on personalized interventions tailored to the pathophysiological features and stages of disease progression. In the early stages of infection, priority should be given to enhancing NETs’ antibacterial efficacy to facilitate rapid pathogen clearance. Conversely, during aseptic inflammation or advanced stages of AS, suppressing excessive NET formation should be prioritized to mitigate tissue damage and CVD progression.

The studies highlighted above demonstrate that NETs play a critical role in the onset and progression of CVDs. A growing body of evidence indicates that TCM and natural products influence the CVD process by specifically targeting and regulating NETs through diverse molecular mechanisms. These findings strongly suggest that TCM and natural products are promising therapeutic agents for CVDs. To date, studies on targeted regulation of NETs by TCM and natural products primarily concentrate on conditions such as MIRI, AS, and PAH. However, research related to HF and AF remains limited. Most existing studies are preclinical and lack extensive long-term clinical trials. Currently, only two NET-targeting compounds have received FDA approval. With a deeper understanding of the molecular mechanisms underlying NET formation and their role in disease progression, the development of more specific NET-targeted drugs becomes feasible [117]. Additionally, many current studies focus solely on the terminal stages of NETs, neglecting their dynamic regulation by TCM and natural products. Furthermore, animal models employed in CVD research exhibit heterogeneity, hindering translation of findings into clinical practice. Researchers must therefore design more rigorous and rational clinical trials to address these gaps. Moreover, continued investigation of NET-related signaling pathways is necessary to clarify the expression patterns of NET-associated genes across different stages of CVD progression. Such studies should also identify key targets through which TCM and natural products exert their regulatory effects on NETs, facilitating the development of novel CVD treatments. Moreover, limited bioavailability, low solubility, poor stability, and the lack of targeted drug-delivery systems restrict the clinical application of TCM and natural products. Consequently, further research is needed to elucidate their mechanisms of action, identify active components, and evaluate their effects on the immune system to ensure safety. Notably, nanotechnology provides an innovative drug-delivery platform, enhancing the bioavailability of TCMs and natural products while minimizing side effects. The design of targeted nanoparticles holds promise as a therapeutic strategy for delivering enzymes or active components capable of degrading NETs. For example, Wu et al. [118] demonstrated that Prussian blue nanoparticles coated with bacterial biomimetic membranes (MPB NPs) could be specifically recognized and internalized by neutrophils. By binding to neutrophils, MPB NPs remove intracellular ROS and inhibit NET formation at lesion sites [118]. Additionally, triptolide-loaded micelles effectively reduce neutrophil and inflammatory monocyte counts in AS plaques of ldlr^-/-^ mice, thereby decreasing NET release [119]. Nevertheless, these studies remain experimental, and clinical translation may take considerable time. Although clinical application of nanomedicines still faces challenges, advancements in nanocarriers for TCM-derived natural products offer innovative and effective approaches for treating CVDs.

In summary, NETs play an essential role in CVD progression. Natural products targeting NETs offer therapeutic potential, including reducing NET formation, promoting NET degradation, modulating intestinal flora composition, and enabling multitarget combination therapies. While many natural products have demonstrated promising outcomes in preclinical trials, their efficacy in treating CVDs lacks validation through clinical trials. Therefore, rigorously designed randomized controlled trials are necessary to confirm the clinical efficacy of natural products in treating CVDs and monitor potential adverse effects. Although research and development of natural drugs for CVD remain at an early stage and face numerous challenges, continuous exploration of natural active ingredients and their mechanisms indicates that TCM and natural products targeting NETs have extensive potential for cardiovascular therapy.

## 6. Conclusions and Future Perspectives

Current treatments for CVD mainly aim to manage symptoms and delay disease progression. However, their effectiveness remains limited and may cause potential adverse effects. TCM and natural products, characterized by multiple components and targets, offer distinct advantages in regulating immune-inflammatory responses and protecting vascular endothelial function. Thus, they provide novel strategies for preventing and treating CVD. Recent research suggests that NETs play critical roles in the pathogenesis of atherosclerosis, heart failure, atrial fibrillation, myocardial ischemia–reperfusion injury, and other cardiovascular conditions. Therefore, NETs represent important potential therapeutic targets in CVD interventions. Preclinical studies and preliminary clinical trials indicate that various active ingredients derived from TCM and natural products alleviate vascular inflammation and tissue injury, influencing CVD pathologies through mechanisms such as inhibiting NET formation, enhancing NET clearance, or regulating associated signaling pathways.

However, many naturally active compounds are limited by factors such as low oral bioavailability and poor targeting capability, hindering their clinical translation. Emerging technologies, including nano-delivery systems and biological carriers, show promise for improving the stability, targeting precision, and therapeutic efficacy of these compounds, thereby facilitating their clinical use. Future studies should further clarify the precise mechanisms of NET involvement in CVD, identify key active components from TCM and natural products that modulate NET-related processes, clarify their molecular targets, and validate their safety and effectiveness through rigorous clinical trials. With a deeper understanding of NET roles in CVDs, along with ongoing advancements in natural product delivery techniques and optimized therapeutic approaches, integrating TCM and natural products into NET-targeted interventions is expected to establish new scientific foundations and clinical pathways for more precise, safe, and effective CVD management.

## Figures and Tables

**Table 1:** Active roles of herbal medicine and traditional Chinese medicine formulas in MIRI by regulating NET formation.

Natural Products and Traditional Chinese Medicine	Experiment Type	NETs-Related Mode of Action	Treatment Effects	Doses/Concentrations	Duration	Reference
Nuanxinkang	in vivo	MPO, NE, CitH3, NLRP3, HMGB1, IL-1β	Nuanxinkang significantly delayed body weight loss, improved cardiac function, reduced myocardial fibrosis, and alleviated oxidative stress	3.5, 7.0, 14.0 g/kg/d	28 days	[35]
Qingxin Jieyu granules	in vivo	ANXA1/FPR2	improves cardiac function and reduces pathological damage in the myocardial tissue of MI rats	1.16, 2.31, 4.62 g/kg/d	5 days	[36]
Formononetin	in vivo	CD36	inhibits platelet activation and NET formation	10, 20, 40 mg/kg/d	7 days	[37]
Dunye Guanxinning	in vivo	IL-1β, AMPK, caspase-1	reduces NETosis, sterile inflammation, cardiomyocyte death, and microthrombosis by blocking neutrophil infiltration	100.8 mg/mg/d	14 days	[39]
Pistachios	in vivo	MPO	inhibit NET formation, thus mitigating myocardial tissue damage	30 mg/kg/d	60 days	[40]
Silibinin	in vivo, in vitro	MPO	alleviating neutrophil infiltration and NET formation	100 mg/mg/d	21 days	[42]
Panaxynol	in vivo, in vitro	MPO, HMGB1, TLR4, NF-κB	reduces pro-inflammatory cytokine levels and serum MPO activity	100, 200, 300 mg/kg/d; 10 mM/mmol/L	30 min	[44]

**Table 2:** The active role of herbal medicine and traditional Chinese medicine formula in AS by regulating the formation of NET.

Natural Products and Traditional Chinese Medicine	Experiment Type	NETs-Related Mode of Action	Treatment Effects	Doses/Concentrations	Duration	References
Dihydrotanshinone I	in vitro	CitH3, MPO, NOX	reduces CitH3 levels during NETosis and inhibits MPO and NOX activities	20 µM	12 h	[58]
Quercetin	in vitro	P2X7R, P38MAPK, NOX2	suppresses LPC-induced NET formation	25 µmol/L	3 h	[61,62]
Ferulic acid	in vitro	CitH3, MPO, CD62p, PAC-1	reduces vasculitis-related symptoms by inhibiting NET release and platelet activation	100 mg/kg/d	24 h	[64]
Paeonol	in vivo, in vitro	CitH3, NLRP3, caspase-1	inhibited NET-induced foam cell inflammation	200, 400 mg/kg/d, 15, 30, 60 µM/L	28 days,4 h	[66]
Modified Taohong Siwu Decoction	in vivo, in vitro	NE, CitH3, CD62P, VCAM-1, ICAM-1	inhibition of endothelial injury and apoptosis by modulating NETs,	11.3 g/kg/d, 0.1 mg, 1, 10 mg/mL	24 h	[70]

**Table 3:** The active role of herbal medicine and traditional Chinese medicine formula in PAH by regulating the formation of NET.

Natural Products and Traditional Chinese Medicine	Experiment Type	NETs-Related Mode of Action	Treatment Effects	Doses/Concentrations	Duration	References
Huoxue Tongluo Formula	in vitro	CitH3, MPO, AKT1, IKK, NF-κB	HXTLF mediated the expression levels of H3Cit and myeloperoxidase (MPO) protein in neutrophils activated by LPS, inhibited NETs formation	2.5%, 5%, 10% HXTLF	4 h	[80]
Qingke Pingchuan granules	in vivo	NOX2, p47phox, ROS	Reduce airway inflammation and lung damage in COPD by suppressing pulmonary NET formation	5, 10 g/kg/d	5 days	[81]
Resveratrol	in vivo, in vitro	CitH3, MPO	Suppresses phosphorylation and enzymatic activity of Src family kinases, mitigating endotoxin-induced lung injury by decreasing MPO levels and neutrophil infiltration	100 mg/kg,10–50 µM	6 h	[82]
Grape seed proanthocyanidin	in vivo	MPO, TNF-α	Decreasing inflammation and MPO expression	10 mL/kg	3 weeks	[83]
Baicalin	in vivo, in vitro	Padi4, CitH3, MPO	Reducing pulmonary artery pressure and vascular remodeling via anti-inflammatory responses	240, 100 mg/kg/d, 25, 50, 100 µM	2 weeks, 2.5 h	[84,85]
Zingerone	in vivo, in vitro	NRF2, ROS	Reduces ROS accumulation and systemic inflammation	25, 50 mg/kg, 5, 25, 50 µM	7 days, 3 h	[86]
Forsythiaside A	in vivo	NOX, PAD4	Reduces NETosis in colon tissues, inhibits PAD4 expression in neutrophils	15, 30, 60 mg/kg	10 days	[87]
Aloperine	in vivo	NOX2, NOX4	Inhibiting NOX2 and NOX4 expression and reducing oxidative stress	25, 50, 100 mg/kg, 5, 25, 50 µM	21 days	[88]

**Table 4:** Active roles of herbal and traditional Chinese medicine formulas in HF and AF by regulating NET formation.

Natural Products and Traditional Chinese Medicine	Experiment Type	NETs-Related Mode of Action	Treatment Effects	Doses/Concentrations	Duration	References
Nuanxinkang	in vivo	NLRP3, HMGB1, IL-1β, CitH3, MPO	enhances cardiac function in mice with ischemic HF by intervening in NET formation	1.65 g/kg	4 weeks	[100]
Resveratrol	in vitro	MPO, ROS	decreases H2O2 levels and alters NE localization	25, 50, 100 µM	30 min	[102]
Geniposide	in vitro	AMPK, PI3K, Akt		25, 50 mg/kg, 1 µM	8 weeks	[103,104]
Celastrol	in vivo, in vitro	NLRP3, Caspase-1, IL-1β	suppression of PMA-induced NET formation in vitro	1 mg/kg/d, 600 nM	28 days, 24 h	[105,106]
Kaempferol	in vivo, in vitro	CitH3, MPO, PAD4	inhibits neutrophil-derived ROS release by suppressing NET formation	25 µmol/L	24 h	[107]

## Data Availability

No new data were created or analyzed in this study. Data sharing is not applicable to this article.
